# Conference Report: SYSTEMS INTEGRATION NEEDS OF U.S. MANUFACTURERS Gaithersburg, MD August 16–17, 1993

**DOI:** 10.6028/jres.099.062

**Published:** 1994

**Authors:** S. L. Stewart, Ginger Pinholster

**Affiliations:** Factory Automation Division, National Institute of Standards and Technology, Gaithersburg, MD 20899-0001; Pinholster Communications, Wilmington, DE 19802

## 1. Introduction

In August 1993, the National Institute of Standards and Technology held a workshop, for industry leaders to address the question of their needs systems integration. These leaders were invited this time because the Institute was on the verge the largest program expansion in its Although the FY 1994 budget had not passed congress at the time of the meeting, the expansion of information technology for manufacturing was very high on the Administration’s list of priorities. This high priority and visibility made early planning an important step in the success of any future program. Accordingly the Institute sought the best possible industrial advice at a time when that advice could have the most impact on program formulation.

Howard Bloom, Chief of the Factory Automation Systems Division and host for the work-shop, welcomed everyone and introduced Arati Prabhakar, Director of the National Institute of Standards and Technology. Dr. Prabhakar noted in her opening remarks that the Institute is entering an era when technology is at the fore of the Administration’s agenda.” Over the next 4 years NIST may well double its current budget of $192.9 million for in-house, laboratory activities. For Fiscal year 1994, the total NIST budget, is expected to jump to about $535.2 million-up from $384 million in 1993.

“AT NIST Today,” Dr. Prabhakar commented, “we’re at the beginning of what I think is going be one of the most exciting times in the history of the Institute.”

In the opening session, prof. Roger Nagel chaired presentation by five speakers to set the context for the workshop:

1. *Overview of U. S. Needs* by Professor James J. Solberg,
2. *Overview of Federal Studies* by Mr. John Meyer,
3. *Agile Manufacturing* by Mr. Rick Dove,
4. *Standards Development* by Ms. Suzanne Olsen, and
5. *Technology Transfer* by Mr. John Leary.

In the panel session, conducted by Ms. S. Jeane Ford, the workshop was divided into group to consider system integration from three perspectives: technology transfer, standards, and technology development. The consensus was that technology transfer and standards were the most important roles for new program direction with a significant, but smaller, role for technology development in conjunction with industry.

When the new initiative did indeed pass, the value of the workshop became evident in specifics of the new program in Systems Integration for Manufacturing Applications (SIMA). Technology transfer was recognized in a major new project for Manufacturing Integration Technology Transfer. The work on the Standard for Exchange of Product Model Data (STEP) was greatly expanded to help meet the standards needs of industry. A new Advanced Manufacturing and Networks Testbed (AMSANT) will support both standards and technology transfer in the new program. Finally, an expanded project for integration will develop new standards for enterprise integration.

The three groups returned from their deliberations with the following specific recommendations and conclusions.

### Technology Transfer Needs

To boost U.S. competitiveness by speeding technology deployment, this working group recommended launching four initiatives:

1. Technology Utilization Self-Assessment Study for small- and medium-sized companies,
2. Technology Transfer Science Study,
3. Technology Transfer Sharing Mechanisms, and
4. Evaluation of the Impact of Government Policies on Technology Transfer.

### Standards-Related Needs

This group felt that current standards-development process needs four critical repairs:

1. A new perspective on the standards-setting process.
2. Better metrics,
3. improved communications between U.S. standards-making groups, and
4. a more effective funding mechanism for standards development.

### Technology-Related Needs

Scalable approaches to systems integration and better metrics for defining success are among the most critical technology-related needs of U.S. manufacturers, according to the third group, which suggested that NIST should:

1. Expand the scope of metrics and lessons learned, providing manufacturers with new tools for rating themselves and setting targets,
2. prepare better metrics for learning and retention, establishing a consistent model of Computer Integrated Manufacturing (CIM) to teach integration technologies,
3. develop a demonstration Virtual Enterprise Testbed at NIST which would allow manufacturers and vendors to plug into the system, to test potential machine tools, software and other technologies, and
4. establish a collaborative program, possibly involving a particular university or a group of universities, to form a Virtual Research and Development Center, thus speeding collaborative developments to market.

## 2. Setting the Context

In the United States, manufacturing generates significant revenue, representing 22 percent of the Gross National Product, and employing 21 million people, or 17 percent of the nation’s total workforce. Noting the new White House Administration’s commitment to a national economic strategy and increased support for U.S. manufacturing, NIST invited top industry experts to discuss their systems integration needs during a workshop on August 16 and 17, 1993. Prof. Roger N. Nagel, Operations Director, Iacocca Institute, and Harvey Wagner Professor of Manufacturing Systems Engineering, Lehigh University, chaired the first session with five speakers to set the context by addressing the question, “Where are we now?”

### Overview of U.S. Needs

“Manufacturing systems,” as defined by Professor James J. Solberg, include every technical, human, and organizational element associated with bringing classes of products into existence, and then disposing of them. Whether it involves linking computer networks or motivating an engineering department to work productively with the accounting department, integrating various systems within a manufacturing setting presents many challenges—which have been addressed by countless studies and management concepts.

Improved systems integration today means “designing and operating manufacturing systems in a coordinated manner, avoiding the consequences of subsystems operating at cross purposes, and avoiding excess cost, lost time, lost quality, and lost opportunity,” according to Professor Solberg. Achieving this goal will require developing new technologies, getting research results into practice faster, and using standards to improve efficiency, he added.

In the past, Professor Solberg said, a relatively stable market made it possible to develop manufacturing processes based on experience, by trial and error. But the current global marketplace demands new models for encapsulating state-of-the-art manufacturing knowledge, as well as sophisticated design tools. More and more often, he added, integration barriers involve human, rather than technical obstacles.

### Overview of Federal Studies

According to Mr. John Meyer, Director of NIST’s Office of Manufacturing Systems, the recent revitalization of the Federal Coordinating Council for Science, Engineering and Technology (FCCSET) reflects “the start of a major transformation of policy related to manufacturing.” (Note: FCCSET was replaced by a cabinet-level National Science and Technology Council (NSTC) after the workshop was held, but the initiatives related to manufacturing are continuing under the NSTC.)

Currently, six Presidential initiatives established under FCCSET address: Advanced Manufacturing Technology (AMT); High Performance Computing and Communications (HPCC); Global Monitoring of the Environment for Environmental Change; Advanced Materials and Processing; Biotecnnology; and Science, Mathematics, Engineering and Technology Education. But Mr. Meyer predicted that this list will soon be reorganized to focus primarily on two super-initiatives: AMT and HPCC.

Before setting up the AMT initiative, FCCSET determined that all U.S. federal agencies in 1994 will spend a total of $1.4 billion on advanced manufacturing technologies representing four general categories: (1) product and process design, (2) manufacturing processes, (3) supporting technologies, and (4) manufacturing infrastructure. Because of FCCSET efforts, an additional $70 million to $80 million worth of funding has been made available for research to support other high-priority technologies that could improve customer satisfaction and help U.S. manufacturers compete more effectively with foreign companies. These technologies include: Intelligent manufacturing cells, integrated design tools, and advanced technology infrastructures.

With a base budget of several billion dollars, the AMT should be launched in 1994 or 1995, Mr. Meyer said, and it is likely to include support for a Clean Car Initiative to develop environmentally benign vehicles.

Until now, the HPCC has focused primarily on scientific and educational applications for high performance computing and networking, but Mr. Meyer said that the initiative will be expanded in 1994 to address many additional areas. Included among the new HPCC applications will be computer-intensive manufacturing problems, such as integrated product and process design through modeling and simulation.

The Department of Defense Manufacturing Systems Strategic Research and Development Plan was prepared to assess the high *manufacturing support* costs associated with purchasing weapons systems. Noting the high pay-back potential of research, the Plan recommended support for the development of new: Integration methodologies, simulation and modeling, and manufacturing engneering support tools.

Collectively, Mr. Meyer noted, all recent studies suggest a need for research and development, in five or six key areas, including: Integration methodologies and tools, standards and frameworks, networking and communications, integration of legacy systems and industry demonstrations of promising concepts such as “agile manufacturing.

### Agile Manufacturing

According to Mr. Rick Dove, President of Paradigm Shift International, the principles of “agile manufacturing” evolved in response to three driving forces in today’s manufacturing environment: Continous change, the need for rapid response, and an evolving definition of quality.

In an environment rife with constant change and increased foreign competition, Mr. Dove explained, U.S. manufacturers most become ever more responsive and flexible. This quality-agility-may be describe as “the ability to thrive in an environment of unpredictable and constant change,” he said.

As increasing globalism changes the U.S. marketplace, he said, the traditional rules of the game (or the “enterprise equation”) have changed. Today, U.S. manufacturers are far more likely to be surprised by a competitor. Thus, they must strive constantly to reduce innovation cycle times, while also fighting the urge to add layers of management, which can make an enterprise more rigid and less responsive to market demands.

Born in chaos theory, which suggests that all events are inherently unpredictable, “agility” is often mistaken for the older concept of “lean manufacturing,” an approach based on efficient practices. But, Mr. Dove said, a truly agile enterprise requires “reconfigurable architecture as a foundation for investments”-whether the systems in question are machine tools, organizational structures, or software integration programs. Today, central planning and hierarchical control no longer work. “I need to be able to reconfigure systems, instead of throwing them out and rebuilding them,” Mr. Dove said.

Technology is important for achieving agility, he added, but people are the real key, since people make decisions, and rapid decision-making is critical in a global marketplace.

### Standards Development

Faced with the rapid proliferation of hardware and software, many major corporations such as General Motors (GM) are trying to build a consistent set of bridges between automation islands by pushing for international standards, explained Ms Suzanne Olsen. Specifically, GM is focusing on the Standard for Exchange of Product Model Data (STEP) as its “strategic direction for product data sharing said Ms. Olsen, a Staff Project Manager for GM’s Technical Center.

While participation in standards development may have been considered a civic duty at one point in time, Ms. Olsen noted, U.S. manufacturers today take part in the standards-setting process because standards clearly help reduce costs over the long-term.

Yet, she said, NIST and industry need to look at the current standards-development process “with a very hard critical eye.” Standards developed through traditional organizations such as the International Organization for Standardization (ISO) and the American National Standards Institute (ANSI) simply take too long to reach the marketplace, she said.

Ms. Olsen concurs with the advice of Ford’s Keith Termaat, who has suggested that manufacturers need to know their customers and deliver a product that achieves better than 95 percent customer satisfaction —while also reducing standards-development time by at least 25 percent.

A proponent of international, rather than *de facto* standards, Ms. Olsen urged NIST to take a leadership role in improving the standards-development process. The U.S. voluntary standards process should not be allowed to stifle efficiency, she said.

### Technology Transfer

In the United States, it takes many years to move new technology into general use, noted Mr. John Leary, citing a study completed by the National Center for Manufacturing Sciences (NCMS). That’s too long, since “it’s only when you ring the cash register that your ideas finally have value and social worth,” said Mr. Leary, Engineering Director for AT&T’s Standards and Global Manufacturing Planning Center.

To maintain a viable middle-class, Mr. Leary said, the United States must embrace advanced manufacturing technologies to achieve faster deployment of new products. Without improvement in manufacturing, he added, “We might end up with a nation where a few smart people will be creating software and designing products, while the rest of us will be flipping hamburgers.”

Various collaborative research initiatives now hold promise for speeding U.S. technology deployment. The NCMS Strategy, for example, provides a framework for joint U.S./Canadian research supported by government and industry. Another collaborative strategy, NIST’s Advanced Technology Program, has been highly effective in cutting technology lag-time. Collaborative ventures invariably increase the amount of market intelligence or know-how around the table, he said, and they reduce financial risks, offering greater leverage for smaller companies.

The key to successful collaboration, Mr. Leary noted, is to bring users and suppliers together—a challenge which has become less complicated since the 1984 Cooperative Research and Development Act eased anti-trust restrictions.

As more and more collaborative ventures are established, Mr. Leary said, NIST should support industry in dealing with intellectual property issues and cultural or “human” obstacles. New technologies, including electronically interfaced information networks, will also be needed to support collaborative ventures.

## 3. Panel Summary: Where Do We Go?

During the NIST workshop, three working groups identified the systems integration needs of U.S. manufacturers in three areas: technology transfer, standards, and technology development. Ms. S. Jeane Ford, the Program Manager of the National PDES Testbed at NIST, led the panel discussion at which the results of the three working groups were presented.

Throughout discussions, participants repeatedly voiced dismay over the nation’s slow technology-transfer process. Not surprisingly, a large number of industry leaders urged NIST to focus most of its resources on support for faster deployment of new technologies. For example, Mr. Michael Kennedy of Texas Instruments echoed the sentiments of other participants when he said: “There are many ways to develop technology quite effectively outside of NIST. NIST should focus instead on technology transfer and standards. The Institute has got to carry the ball in those areas.”

NIST should also lead the charge in reengineering the standards-development process, workshop participants said. It would be appropriate now for NIST to take the lead in pushing for better standards to support U.S. manufacturing.

Among the new technologies seen as critical tor U.S. manufacturers, participants identified numerous integration methodologies such as scalable approaches to systems integration, as well as improved metrics.

The participants’ recommendations are summarized in the following sections.

### Technology Transfer Needs

To boost U.S. competitiveness by speeding technology deployment, Mr. Peter N. Butenhoff reported, NIST should launch four initiatives.

1. Technology Utilization Self-Assessment Study for small- and medium-sized companies,
2. Technology Transfer Science Study,
3. Technology Transfer Sharing Mechanisms, and
4. Evaluation of the Impact of Government Policies on Technology Transfer.

Designed to help smaller companies determine their technology transfer needs in the face of increasingly fierce competition, the Technology Utilization Self-Assessment Study would include a “self-evaluation checklist” as well as a collection of “failure stories” illustrating the consequences of technological neglect, said Mr. Butenhoff, President of the Textile/Clothing Technology Corp. (TC2). Examples of “best practices,” training laboratories for schools, and other manufacturing extension services could also be a part of the Self-Assessment Study.

Noting that “technology transfer” is a poorly defined process, Mr. Ronald Dick and others proposed a “Technology Transfer Science Study” to clarify the issue. Among other objectives, the Study would identify companies achieving technology transfer, develop a set of business cases related to technology deployment, and establish a process for applying new technology to commercial products, said Mr. Dick, Technical Director for IMAR.

Workshop participants also called for additional Technology Transfer Sharing Mechan.sms. Specifically, the group urged NIST to establish a computer support system featuring: Electronic networking to industry and universities, an on-line database of abstracts describing technology, and user-friendly search techniques. By accepting a leadership role in commercialization endeavors and by organizing national symposia on successful transfer cases, NIST could provide additional support for rapid technology deployment

Finally Mr. Butenhoff said, NIST’s charter should be broadened to include research of business practices and cultures. A broader mission statement would allow the Institute to conduct an Evaluation of Government Policies on Technology Transfer, to determine which small manufacturing environments are critical to the entire U.S. economy, and to evaluate tex incentives for promoting technological advances.

### Standards-Related Needs

According to Mr. Jack White, new standards succeed for three reasons: they are demanded by major user, they are driven by clear business needs, and they are supported by many vendors, or at least by a few market leaders. Whether they bubble up from a grassroots movement, or fall from a top-down development, Program, both *de facto* and proprietary standard must be clearly needed to be successful, said Mr. White of the Industrial Technology Institute

Unfortunately, he said, the United States’ current standards-development process is broken, and it will require four critical repairs: (1) a new perspective on the standards-setting process, (2) better metrics, (3) improved communications between U.S. standards-making groups, and (4) a more effective funding mechanism for standards development.

“NIST should lead the charge to re-engineer the critical processes involved in developing standards,” Mr. White said. This effort should involve many different groups, including industry leaders, vendors, and consortia directors. Ultimately, the re-engineering effort should result in a set of “best practices” for standards-making. To sell the new approach to potential users, NIST could parlay its reputation for excellence in manufacturing support, said Mr. Michael Kennedy of Texas Instruments.

Improved metrics are essential for measuring the progress and quality of NIST’s re-engineered standards-development process, Mr. White added. At the same time, NIST will need to maintain new electronic repositories for information on U.S. and global standards efforts. Funding could be provided through a new Standards Development Program, which would focus part of the efforts of the Advanced Technology Program (ATP) and the Advanced Manufacturing Systems and Networking Testbed (AMSANT) on standards and implementation. ATP may be contacted for more information by telephone at 1-800-287-3863 or by email at atp@micf.nist.gov or by fax at 1-301-926-9524. For more information about AMSANT or any other FASD programs, please see contact information at the end of this article.

Like Suzanne Olsen, the standards-related needs group suggested that NIST adopt the guidelines established by Ford Motor Company, which strives to reduce standards deployment time by at least 25 percent, while reducing internal expenses by 20 percent.

“Our group is urging NIST to become pro-active about reengineering standards development in this country “Mr. White said. “We’re also strongly in favor of eliminating redundancy in the standards-making process-including redundant organizations. In the standards game, less is definitely best.”

### Technology-Related Needs

Scalable approaches to systems integration and better metrics for defining success are among the most critical technology-related needs of U.S. manufacturers, according to a group led by Dr. Michael C. Smith of Science Applications International Corp.

To support manufacturing in the 21st Century, Dr. Smith’s group identified these and 37 other specific technology requirements, representing six general categories:

1. Integration Methodologies,
2. Business Models,
3. Interoperable Tools,
4. Active Learning and Feedback,
5. Human Interfaces, and
6. Education and Training.

Meeting industry’s future technology needs will require NIST to undertake four basic development activities, said Mr. Gary K. Conkol of Cleveland Advanced Manufacturing Program. Specifically, Mr. Conkol and others suggested that NIST should:

1. Expand the scope of metrics and lessons learned, providing manufacturers with new tools for rating themselves and setting targets;
2. Prepare better metrics for learning and retention, establishing a consistent model of CIM to teach integration technologies;
3. Develop a demonstration Virtual Enterprise Testbed at NIST which would allow manufacturers and vendors to *plug into* the system, to test potential machine tools, software and other technologies; and
4. Establish a collaborative program, possibly involving a particular university or a group of universities, to form a Virtual Research and Development Center, thus speeding collaborative developments to market.

After the workshop, Tom Rhyne, a workshop participant, took the extra time to write and contribute his personal summary of what NIST should do in both the shorter- and longer-term. Here are his comments with special emphasis on standards. From a tactical (shorter-term) point of view:

- Provide full-time technical experts to participate in support of volunteer participants in key standards activities.
- Serve as a neutral site for demonstration projects involving proposed standards. Such demonstrations can identify strengths or weakness in the proposals. For U.S. proposals, the fact that functionality has been proven by demonstration will strengthen the likelihood of adoption. For non-U.S. proposals, the demonstrations will help decide the appropriate U.S. position on the proposal as well as serve as an initial start to the technology transfer and commercialization of the new standard, if it is adopted.
- Support the continued presence of U.S. experts in leadership positions within critically important standards activities (travel support, part-time support for labor).
- Enhance awareness within U.S. industries of current and emerging standards activities which may have impact on their industrial activities and competitiveness.
- Assure commercial vendors within the U.S. that emerging standards are worthy of their investment, thereby helping to “jump-start” them into making those investments. (Committing limited development resources to an emerging standard is always a very risky decision.)

From strategic point of view:

- Review international standards activities to rank their importance to current and future U.S. industrial competitiveness, and become proactive in the high-priority areas, as for example, by proposing new STEP application protocols (AP’s).
- Make certain that U.S. participation in those standards activities marked as critical to U.S. interests is effective and solid. (I believe that the United States can no longer accept a volunteer catch-as-catch-can approach to participation in key standards activities. Instead, we need a well selected, properly supported team of participants, and NIST, in the Department, of Commerce, needs to assume a clear leadership position in recruiting, training, guiding, supporting those individuals.)
- Expand the demonstration and proof-of-concept laboratory proposed above to invole pilot projects which unite potential vendors and users of proposed standards in activities which (a) provide technical backing to U.S. standards proposals and (b) serve as accelerators to commercial deployment of emerging standard within the United States.
- Seek ways to deploy advanced technologies in support of critical standards process. (Using semi-automated information modeling technology to help accelerate the STEP AP interpretation process is an example.)
- Seek opportunities to move *de facto* standards activities into the formal international standards pipeline.
- Provide assurance that draft international standards and even drafts for comment arc properly evaluated by appropriate experts within the United States, considering both technical merit and potential impact on U.S. industry. Thereafter, provide assurance that appropriate U.S. positions are produced and forwarded to the adopting body.

For more information, please contact the Factory Automation Systems Division, Building 220, Room A-127, NIST, Gaithersburg, MD 20899-0001 This report and additional material about the work of the division are available electronically at http://elib.cme.nist.gov/fasd/fasdhome.html or ftp://ftp.cme.nist.gov/pub. The division office can be reached by Telephone at 1-301-975-3508 or fax at. 1-301-258-9749.

## 3. Appendix A. Agenda

**Welcome and Introduction**

Dr. Arati Prabhakar, Director, NIST
Mr. Howard M. Bloom, Chief, FASD

**Session 1: Invited Presentations**

Chair Prof. Roger N. Nagel
  Operations Director, Iacocca Institute
  Harvey Wagner Professor of Manufacturing
  Systems, Lehigh University

Overview of U.S. Needs

Prof. James J. Solberg
  Director, Engineering Research Center,
  Purdue University

Overview of Federal Studies

Mr. John Meyer
  Director, Office of Manufacturing Programs, NIST

Agile Manufacturing

Mr. Rick Dove
President, Paradigm Shift International

Standards Development Needs of U.S. Industry

Ms. Suzanne Olsen
  Staff Project Engineer, General Motors
  Technical Center

Technology Transfer Needs of U.S. Industry

Mr. John Leary
  AT&T Technologies, Inc. and ANSI CIM
  Standards Board

**Session 2: Discussion Groups**

Group 1: Technology-Related Needs
Discussion Leader: Michael C. Smith, Science Applications Intl. Corp.
Reporter: Gary K. Conkol, Cleveland Advanced Manufacturing Program
Group 2: Standards-Related Needs
Discussion Leader: Jack White, Industrial Technology Institute Reporter: Michael Kennedy, Texas Instruments
Group 3: Technology Transfer Needs
Discussion Leader: Peter N. Butenhoff, President, Textile/Clothing Technology Corp.
Reporter: Ronald L. Dick, Technical Director IMAR

**Session 3: Discussion Group Results**

Chair: S. Jeane Ford, Program Manager, National PDES Testbed, NIST
Reports of Small Group Discussions
  Presented by reporters
Panel Discussion/Clarification of Industry Needs
  Panel of discussion leaders and reporters from each small group.
Wrapup and Agreement on Time Table for Subsequent Steps
  S. Jeane Ford

**Closing**

Howard M. Bloom

## 4. Appendix B. Participants

**Mr. Arlan Andrews**, Sandia National Laboratory

**Mr. Ah Bahroloomi**, NAO Manufacturing Information Systems, General Motors

**Mr. Howard Bloom**, Chief, Factory Automation Systems Division, NIST

**Mr. Randolph L. Bumette**, Director, Merchandise Planning and Quick Response, Mercantile Stores Company, Inc.

**Mr. Peter N. Butenhoff**, President, Textile/Clothing Technology Corp. (TC2)

**Mr. Gary K. Conkol**, Cleveland Advanced Manufacturing Program

**Mr. John Decaire**, Acting Director of Technology, National Center for Manufacturing Sciences

**Mr. Ronald L. Dick**, Technical Director, IMAR

**Mr. Rick Dove**, President, Paradigm Shift International

**Mr. Bob Finkelstein**, President, Robotic Technology, Inc.

**Ms. S. Jeane Ford**, Program Manager, National PDES Testbed, NIST

**Mr. Glenn Hollowell**, SEMATECH

**Mr. Joe Iseman**, Innovative Technologies

**Mr. Michael Kennedy**, Texas Instruments

**Mr. Robert Kiggans**, General Manager, PDES, Inc.

**Mr. Tim Lacoss**, Mechanical Engineer Advanced Technologies Design Division, Watervliet Arsenal

**Mr. John Leary**, Engineering Director, Standards and Global Manufacturing Planning Center, AT&T Technologies, Inc.

**Mr. Tom Mahoney**, Manufacturing Studies Board, National Research Council

**Mr. John Meyer**, Director, Office of Manufacturing Systems, NIST

**Prof. Roger N. Nagel**, Operations Director, Iacocca Institute, Harvey Wagner Professor of Manufacturing Systems Engineering, Lehigh University

**Ms. Suzanne Olsen**, Staff Project Engineer, General Motors Technical Center

**Dr. Arati Prabhakar**, Director, NIST

**Mr. Tom Rhyne**, MCC/Atlas Standards Laboratories

**Mr. Simon Schurr**, Lehigh University

**Dr. Michael C. Smith**, Science Applications Intl. Corp.

**Mr. Neil Snodgrass**, DACOM

**Prof. James J. Solberg**, Director, Engineering Research Center for Intelligent Manufacturing Systems, Purdue University

**Dr. Seiden Stewart**, Factory Automation Systems Division, NIST

**Mr. Art Sullivan**, Draper Laboratory

